# Prevalence of bovine tuberculosis in cattle, goats, and camels of traditional livestock raising communities in Eritrea

**DOI:** 10.1186/s12917-018-1397-0

**Published:** 2018-03-07

**Authors:** Michael K. Ghebremariam, A. L. Michel, J. C. M. Vernooij, M. Nielen, V. P. M. G. Rutten

**Affiliations:** 1 Department of Infectious Diseases and Immunology, Faculty of Veterinary Medicine, Utrecht University, Utrecht, The Netherlands; 2 Department of Veterinary Sciences, Hamelmalo Agricultural College, Keren, Eritrea; 3 Department of Veterinary Tropical Diseases, Bovine Tuberculosis and Brucellosis Research Programme, Faculty of Veterinary Science, University of Pretoria, Pretoria, South Africa; 4 Research Associate at the National Zoological Gardens of South Africa, Pretoria, South Africa; 5 Department of Farm Animal Health, Faculty of Veterinary Medicine, Utrecht University, Utrecht, The Netherlands

**Keywords:** Bovine tuberculosis, Camels, Eritrea, Goats, Mixed crop-livestock system, Pastoral system, Single intradermal comparative tuberculin test (SICTT)

## Abstract

**Background:**

The aim of the current study was to assess the prevalence of bovine tuberculosis (BTB) in cattle, goats, and camels, and its zoonotic potential within the traditional livestock raising communities in four regions of Eritrea. The Single Intradermal Comparative Tuberculin Test (SICTT) as indicator of *M. bovis* infection was conducted on 1077 cattle, 876 goats, and 195 camels. To elucidate possible risk factors for BTB transmission between animals and its potential zoonotic implication, questionnaire based face-to-face interviews were conducted in households of which 232 raised cattle, 128 goats, and 29 camels.

**Results:**

The results of the SCITT were interpreted using the OIE standard (> 4 mm cut-off) for positive responses. In cattle, individual animal (*n* = 1077) and herd (*n* = 413) prevalences were 1.2% (*n* = 13) [Confidence Interval (CI) 95% CI, 1.0–1.3%] and 3.2% (*n* = 13) (95% CI, 3.0–3.4%), respectively. In goats (*n* = 876), none of the animals was positive. In camels, individual animal (*n* = 195) and herd (*n* = 70), BTB prevalences were 1.5% (*n* = 3) (95% CI,1.4–1.6%) and 2.9(*n* = 2) (95% CI, 0.9–4.6%), respectively. Overall, male animals were more at risk (OR = 2.6; 95% CI:1.0–8.7) when compared to females. Sharing of water points, introduction of new animals into herds and migration of animals over large distances were common events that may contribute to intra and inter-species transmission of BTB. Consumption of raw milk, lack of BTB transmission awareness, and low levels of education were common in the farming communities.

**Conclusion:**

The current study highlighted a low prevalence of *M. bovis* in cattle, goats and camels in extensive traditional livestock in Eritrea. Despite this, the spatial distribution of affected animals across most of the sampled regions and consumption of unpasteurized milk warrants surveillance, cautious and timely control measures for the disease.

**Electronic supplementary material:**

The online version of this article (10.1186/s12917-018-1397-0) contains supplementary material, which is available to authorized users.

## Background

Bovine tuberculosis (BTB) is a chronic bacterial disease caused by *Mycobacterium bovis* (*M. bovis*), a member of the group known as *Mycobacterium tuberculosis complex* (MTC), that has a wide host range. It predominantly affects cattle, but also other domesticated species, like goats [1–4], and camels [5, 6], as well as many wildlife species [7, 8]. In general, in traditional livestock raising systems, cattle and goats are often herded together and watering points are shared by many animal species. Such livestock husbandry and management systems can be an important risk factor for animal-to-animal, animal-to-human, human-to-animal, and human-to-human *M. bovis* transmission [9–13].

*M. bovis* infected animals, as indicated by SICTT, were present in the ‘intensive’ dairy husbandry system of the major milk producing regions in Eritrea [14, 15]. Besides, the presence of *M. bovis* was confirmed by bacterial culture and molecular diagnostic tools from bovine tissues collected at the Asmara slaughterhouse (Ghebremariam, unpublished data). However, the BTB status was never studied in the extensive traditional livestock (pastoral and mixed crop-livestock) system, which comprises the largest percentage of the livestock population (approx. > 99.9%) [14]. In neighbouring Ethiopia, with similar agricultural settings, BTB was reported to be prevalent in the ‘intensive’ dairy cattle in different studies (11.6% and 22.1%, respectively) [16, 17], as well as in cattle in the traditional extensive livestock husbandry system (8.2% and 11%, respectively) [16–18].

**Fig. 1:**
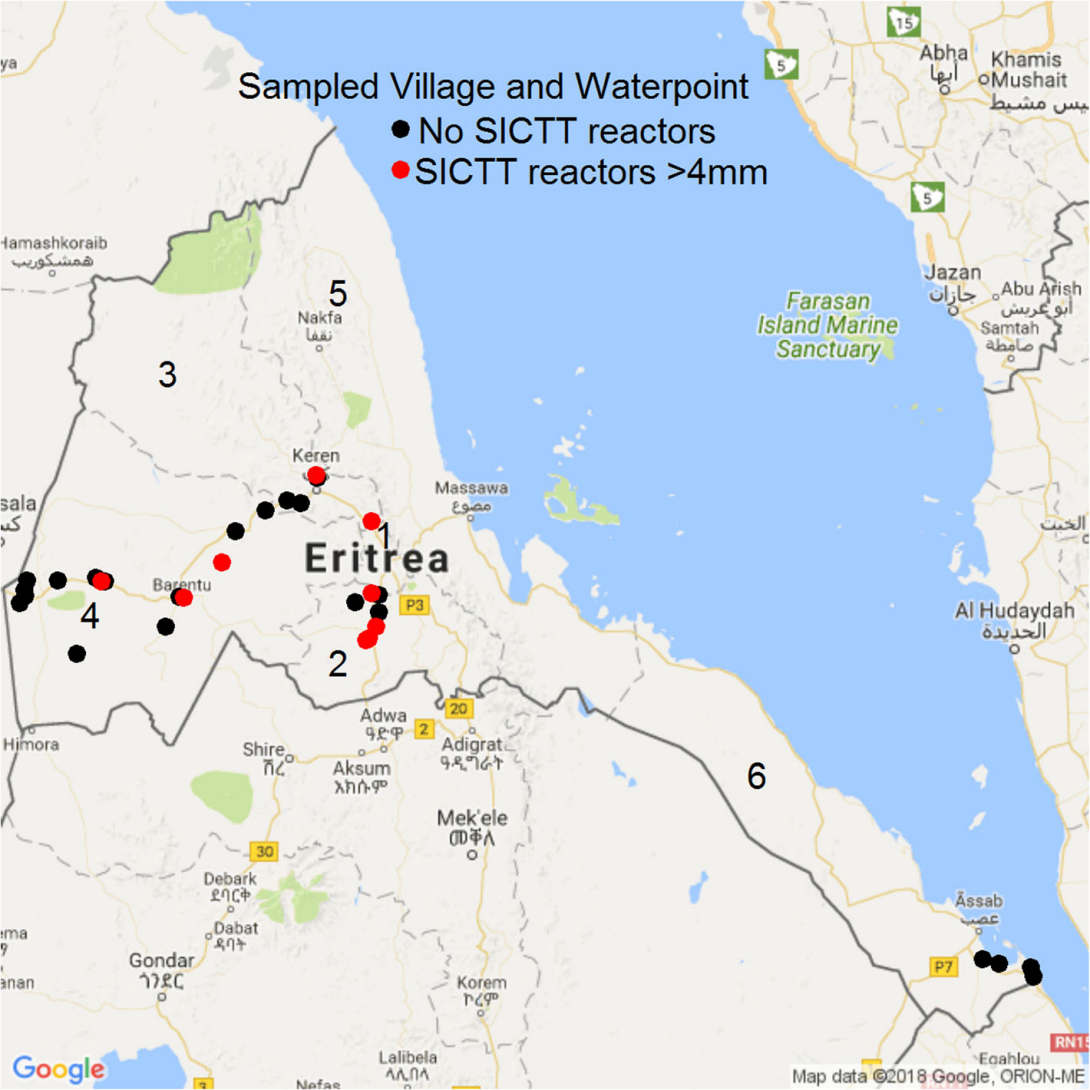
Map of Eritrea depicting the study areas (*n* = 31) and water points (*n* = 4, shared by animals from 11 study areas), having skin test reactors with > 4 mm-cut-off (red dots) and those with no reactors using > 4 mm (black dots) in the selected study areas within the traditional livestock husbandry system in Eritrea. The numbers (1–6) indicated on the map show the six administrative regions of Eritrea (1 = Maekel; 2 = Debub; 3 = Anseba; 4 = Gash Barka; 5 = Northern Red Sea; 6 = Southern Red Sea) (Adapted using: Loecher and Ropkins [54]. RgoogleMaps and loa: Unleashing R Graphics Power on Map Tiles. Journal of Statistical Software 63(4), 1–18. URL http://www.jstatsoft.org/v63/i04/)

In developing countries, especially in rural settings, where dwelling areas may be shared between humans and animals, humans may become infected. This may occur through the inhalation of cough sprays released by chronic coughing animals [9, 12], or/and by drinking raw milk from infected animals [1, 19, 20]. The aim of the current study was to assess the prevalence of BTB in cattle, goats, and camels, and its zoonotic potential within the traditional livestock raising communities in the four regions (Debub, Anseba, Gash Barka, and Southern Red Sea) in Eritrea that share borders with at least one of the neighbouring countries (Fig. 1).

## Methods

### Study population and sample size determination

We conducted a BTB prevalence study using multistage sampling in Debub, Anseba, Gash Barka, and Southern Red Sea regions of Eritrea (Fig. 1), from October to November 2013, and September to December 2014. First, we selected the regions with the target species of animals (cattle, goats and camels or at least two of the species) and those sharing common borders with neighbouring countries (Ethiopia, Sudan, Djibouti) (Fig. 1). For the second stage official lists of sub-regions, including villages that were connected by paved and dry weather roads were used to randomly select villages where two or three of the target species were present. Participation of livestock owners/herdsmen present at the testing sites was voluntary, and only animals of those who gave their consent to participate were recruited for the study. Convenience sampling was applied by selecting animals that could be caught and restrained for testing, and if possible, the questionnaire was concurrently completed by the owners/herdsmen. In the Debub region, as camel raising is not a common practice, hence camels were not included in the study there. Since the prevalence of BTB in the extensive livestock production system in Eritrea was unknown, required sample sizes were determined considering the livestock population size per species (data provided by the Ministry of Agriculture, not shown) of the selected sub-region and assuming 3% prevalence as found in a similar study in extensive traditional livestock farming in Ethiopia [21], and 95% level of significance, using the WIN EPISCOPE 2.0, veterinary epidemiological computer software.

Outcomes of calculations dictated a minimum of 99 animals from each species per study region to be tested to find at least one positive animal. The precision for the estimated prevalence assuming 3% prevalence and the calculated sample size is between 0.6 and 8.5%. But as animals are clustered within herds we aimed for a higher number of cattle and goats by selecting 3% of each herd per species for testing. All the target species of animals were sampled up to a herd size of 3 and at least up to a convenient higher number for larger farms.

That is, in the presence of three or less animals in a herd of a given species (cattle, goats or camels), the whole herd was subjected to testing. However, although the sample size (3% prevalence) requires 99 animals to be tested per region, it was not possible to reach that number at all times. Particularly, camels were less abundant and not represented in all herds.

The test sites were water points (*n* = 4, where animals from 11 study areas were tested), villages (*n* = 21), livestock gathering (resting) (*n* = 4 sites) and grazing areas (*n* = 6 sites) as they provide frequent opportunities for inter-and intra-species contact.

Geographic coordinates were registered at the test sites (areas) by the global positioning system (GPS) (Fig. 1). Information relating to age, sex, region, sub-regions, and study areas was recorded for all the tested cattle, goats and camels (Additional file 1). In the testing areas animals were restrained by manually handling them using 3–4 men, and were ear tagged for identification purposes.

### Questionnaire survey among livestock owners

In total, 232 cattle, 128 goats, and 29 camel owners, who presented their animals for tuberculin testing, were interviewed face-to-face using a standardized questionnaire consisting of both open and closed questions, based on their consent to voluntary participation (Additional files 2, 3, 4 and 5). The questionnaire included potential risk factors (variables) for *M. bovis* transmission amongst animals, and between animals and humans: housing of animals, source of water for the animals, sharing of water points, livestock migration, presence of wild animals, farmers’ BTB transmission awareness, raw milk consumption habits, and farmers’ level of education, etc. The questionnaires were translated from English into two local languages (Tigre and Tigrigna) that are widely spoken in the study areas. Initially, the questionnaire was pre-tested by 10 randomly selected households in one of the villages to verify if the questions were clearly understood by the respondents. Subsequently the questionnaire was fine-tuned (ambiguous/misleading questions were corrected or omitted) and used. Administrators of the selected sub-regions, villages, including village elders, were informed of the testing program through regional administration offices to make them aware of and sensitize them to it. The respondents were informed about the confidentiality of data collected through the questionnaire and the interviews were conducted upon the consent of the respondents. It was not possible to interview all the farmers that presented their animals for testing, since at some moments, sites were too crowded to allow timely completion of the questionnaires, and the farmers were leaving the sites.

### Intradermal tuberculin skin testing

The skin testing was conducted according to the OIE [22] standard. Briefly, two sites on the left side of the mid-neck in cattle, right and left neck in goats, and upper and lower neck, closer to the shoulder, in camels, were shaved 12 to15cm apart. At the indicated sites skin thickness was measured with a ‘Vernier calliper’ and recorded, and the sites were injected with 0.1 ml containing 2500 IU avian PPD (Prionics, Lelystad, The Netherlands) and 0.1 ml of 3000 IU bovine PPD (Prionics, Lelystad, The Netherlands) using McLintock pre-set automatic syringes. Correct injection was confirmed by palpation of a small pea-like swelling at each site of injection.

Seventy-two hours after inoculation, the skinfold thicknesses at the injection sites were re-measured by the same operator and with the calliper used before. The readings were interpreted using the standard (> 4 mm cut-off; OIE) method [22]. An animal was considered positive if the differential increase in skin thickness between the bovine and avian injection was greater than 4 mm, inconclusive when the reaction difference was 1–4 mm, and negative when the bovine reaction was less or equal to the avian reaction in the absence of any clinical signs at the injection site. For the logistic regression analysis, we considered the inclusive results as negatives. As an incentive for participation by the owners, their animals were treated for internal and external parasites with ivermectin (anthelmintics) following the reading of the results. Though we have used standard interpretation of the results throughout the manuscript, we have included severe (> 2 mm cut-off) method for comparison with other studies (Additional files 6 and 7). According to the severe method, an animal was considered positive if the bovine minus the avian reaction was greater than 2 mm.

### Data analysis

Data were entered in an Excel spreadsheet and then exported to SPSS IBM version 20 for analysis and all the analyses were conducted using this statistical package. Descriptive analysis was conducted at both individual animal and herd levels. Due to repeated samples within herds a mixed effect logistic regression model would be most appropriate to account for this dependency. A considerable number of farms have only one sample which causes problems in the estimation of the farm effects (farm level is the level of the only sampled animal). Secondly the outcome is binary (pos/neg per animal) which means that with 3 animals tested in a herd only a few levels for estimation maybe observed (0, 33, 66, 100% positive). The expected prevalence is 3%, so at farm level the prevalence will be mostly 0%. Therefore, we used an ordinary logistic regression model to investigate the relationship between the potential risk factors and outcomes. First, in univariable analysis, the measure of association between each of the potential risk factors and BTB (skin test positive score as a parameter), was examined for each factor individually and evaluated for statistical significance using a Fisher’s Exact test for independence; secondly, multivariable logistic regression was applied to estimate the measures of associations between the potential risk factors (species, age, and sex of the tested animals) and the outcome were tested resulting in odds ratios (OR). The outcome of all statistical analyses were individual animal and herd level binary outcomes. A herd was considered positive if it had at least one skin test positive reactor using the > 4 mm cut-off.

## Results

The Single Intradermal Comparative Tuberculin Test (SICTT) was conducted in 1077 cattle, median mean herd size 3.00 (range: 1–31), from 36 study areas and 11 sub-regions, 876 goats, median herd size 4.00 (range:1–15) from 27 study areas and 9 sub-regions, and 195 camels, median herd size 6.0 (range:1–11) from 16 study areas and 7 sub-regions (Table 1). In total, 413 cattle, 243 goat, and 70 camel herds were tested (Table 2). Overall, females accounted for 71% of animals tested (*n* = 764) in cattle, 95.5% (*n* = 838) in goats, and 75% (*n* = 146) in camels (Table 3).

**Table 1:** Number (and herd) of skin tested cattle, goats and camels, and number (herd) of reactors at region, sub-region and study areas levels using the standard (> 4 mm cut-off) method in the selected study areas within the traditional livestock husbandry system in Eritrea. ‘0’ = zero animals tested from zero herds. NA = not applicable

Regions	Sub-region	Study Areas	Tested cattle(herd)	Reactor cattle (herd)	Tested goats (herds)	Reactor goats (herd)	Tested camels (herds)	Reactor camels (herd)
				> 4 mm		> 4 mm		> 4 mm
Debub	Dbarwa	DRW1	61(20)	0(0)	60(13)	0(0)	NA	NA
		DRW2	44(29)	3(3)	15(6)	0(0)	NA	NA
		DRW3	38(26)	0(0)	17(7)	0(0)	NA	NA
		DRW4	48(15)	0(0)	25(6)	0(0)	NA	NA
	Total		191 (90)	3(3)	117 (32)	0(0)	NA	NA
	Mendefera	MFR1	104(35)	4(4)	38(7)	0(0)	NA	NA
		MFR22	52(23)	1(1)	16(5)	0(0)	NA	NA
		MFR3	38(15)	1(1)	0(0)	–	NA	NA
	Total		194 (73)	6(6)	54(12)	0(0)	NA	NA
Anseba	Hagaz	HAZ1	3(2)	0(0)	15(2)	0(0)	0	–
		HAZ2	12(2)	0(0)	10(1)	0(0)	1(1)	0(0)
		HAZ3	3(1)	0(0)	0	–	0	–
		HAZ4	8(2)	0(0)	0	–	0	–
		HAZ5	0	–	13(2)	0(0)	10(1)	0(0)
		HAZ6	0	–	3(1)	0(0)	0	–
		HAZ7	0	–	5(1)	0(0)	0	–
	Total		26(7)	0(0)	46(7)	0(0)	11(2)	0(0)
	Hamelmalo	HAM1	14(6)	0(0)	0	0(0)	0	–
		HAM2	1(1)	0(0)	0	0(0)	0	–
		HAM3	61(27)	0(0)	37(7)	0(0)	10(3)	3(2)
		HAM4	45(11)	0(0)	11(2)	0(0)	0	–
		HAM5	0(0)	–	0(0)	–	0	–
	Total		121(45)	0(0)	48(9)	0(0)	10(3)	3(2)
	Adi-Tekelezan	ATK1	72(35)	1(1)	39(9)	0(0)	0	–
	Total		72(35)	1(1)	39(9)	0(0)	0	–
Gash Barka	Barentu	BAR1	89(32)	1(1)	0	–	0	–
		BAR2	28(6)	0 (0)	7(2)	0(0)	0	–
		BAR3	6(4)	0(0)	49(13)	0(0	13(10)	0(0)
		BAR4	22(10)	0 (0)	72(18)	0(0)	2(2)	0 (0)
	Total		145(52)	1(1)	128(33)	0(0)	15(12)	0(0)
	Tessenei	TES1	28(12)	0 (0)	163(70)	0(0	1(1)	0 (0)
		TES2	11(4)	0 (0)	22(9)	0(0)	0	–
		TES3	0	–	0	–	71(20)	0 (0)
		TES4	30(10)	0 (0)	55(21)	0(0)	1(1)	0 (0)
		TES5	15(5)	0(0)	10(5)	0(0)	0(0)	–
	Total		84(31)	0(0)	250(105)	0(0)	73(22)	0(0)
	Hykota	HYK1	17(5)	0(0)	0	–	0	–
		HYK2	16(8)	0 (0)	0	–	1(1)	0 (0)
		HYK3	11(11)	1(1)	0	–	10(2)	0(0)
		HYK4	8(1)	0(0)	0	–	0	–
		HYK5	28(8)	0 (0)	0	–	0	–
		HYK6	14(14)	0 (0)	0	–	48 (6)	0 (0)
		HYK7	31(1)	0(0)	0	–	0	–
		HYK8	28(1)	0(0)	0	–	0	–
	Total		153(49)	1(1)	0	–	59(9)	0(0)
	Mogolo	MOG1	46(9)	1(1)	25(2)	0(0)	11(8)	0 (0)
	Total		46(9)	1(1)	25(2)	0(0)	11(8)	0(0)
	Akurdet	AKU1	25(15)	0 (0)	0	–	0	–
		AKU2	18(5)	0(0)	0	–	0	–
	Total		43(20)	0(0)	0(	–	0	–
Southern Red Sea	Debub Dankalia	DANK1	0	–	17(4)	0(0)	3(3)	0 (0)
		DANK2	0	–	10(1)	0(0)	2(2)	0 (0)
		DANK3	0	–	54(13)	0(0)	0	–
		DANK4	2(2)	0(0)	35(10))	0(0)	0	–
		DANK5	0	–	53(6)	0(0)	11(9)	0(0)
	Total		2(2)	0(0)	169(34)	0(0)	16(14)	0(0)
	Grand Total		1077(413)	13(13)	876(243)	0(0)	195(70)	3(2)

**Table 2:** BTB prevalence in cattle, goats and camels at individual animal and herd levels within the traditional livestock husbandry system in the study regions using standard cut-off (> 4 mm). NA = not applicable

Number and herds of cattle, goats and camels tested	Anseba	Debub	Gash Barka	Southern Red Sea	Overall
Number (%) of cattle	219 (20.3)	385 (35.7)	471 (43.7)	2 (0.2)	1077 (100)
Number (%) of goats	133 (15.2)	171 (19.5)	403 (46)	169 (19.3)	876 (100)
Number (%) of camels	21 (10.8)	NA	158 (81.0)	16 (8.2)	195 (100)
Total number (%) tested/region	373 (17.4)	556 (25.9)	1032 (48)	187 (8.7)	2148 (100)
Individual animal Prevalence (%)					
Cattle	1 (0.5)	9 (2.3)	3 (0.6)	0 (0.0)	13 (1.2)
Goats	0 (0.0)	0 (0.0)	0 (0.0)	0 (0.0)	0 (0.0)
Camels	3 (13.6)	NA	0 (0.0)	0 (0.0)	3 (1.5)
Herds of cattle, goats and camels tested					
Herds (%) of cattle	87 (21.1)	163 (39.5)	161 (38.9)	2 (0.5)	413 (100)
Herds (%) of goats	25 (10.3)	44 (18.1)	140 (57.6)	34 (14.0)	243 (100)
Herds (%) of camels	6 (8.3)	0 (0.0)	50 (72.2)	14 (19.4)	70 (100)
Herd prevalence (%)					
Cattle	1 (1.2)	9 (5.5)	3 (1.9)	0 (0.0)	13 (3.2)
Goats	0 (0.0)	0 (0.0)	0 (0.0)	0 (0.0)	0 (0.0)
Camels	2 (33.3)	NA	0 (0.0)	0 (0.0)	2 (2.9)

**Table 3:** BTB prevalence as associated to sex in cattle, camel, and goats within the traditional livestock husbandry system using > 4 mm

Status	*P*-value	Female	Male
		764 (79.9)	313 (29.1)
Cattle: > 4 mm cut-off	(0.089)	Number (%)	Number (%)
Inconclusive		146 (19.1)	67 (21.4)
Negative		612 (80.1)	239 (76.4)
Positive		6 (0.8)	7 (2.2)
Camels: > 4 mm cut-off	(0.053)	146 (74.9)	49 (25.1)
Inconclusive		22 (15.1)	15 (30.6)
Negative		122 (83.0)	33 (67.4)
Positive		2 (1.4)	1 (2.0)

### Tuberculin reactors in cattle, goats and camels at individual and herd levels

#### Cattle

Results of the SICTT are shown in Table 2. The overall individual animal and herd prevalences, using the standard method were 1.2% (13/1077) [Confidence Interval (CI), 95% CI, 1.1–1.3%] and 3.2% (13/413), (95% CI, 3.0–3.4%), respectively. Within the cattle rearing villages, 22% (8/36) of the herds had positive reactors (Tables 1 and 2). Whereas, using the severe method (> 2 mm cut-off), the individual and herd prevalences were 5.5% (59/1077) and 13% (54/413), respectively, and more (58%; 21/36) villages had at least one reactor cattle when compared with the standard method (22%; 8/36) (Additional files 6 and 7).

#### Goats

No reactor animals were detected in goats in all the tested regions using the standard method (> 4 mm cut-off). Using the severe method (> 2 mm cut-off), 2.2% (19/876) of the individual goats and 4.9% (12/243) of the herds were reactors. With this method about 30% (7/27) of villages had at least one reactor goat (Additional files 6 and 7).

#### Camels

In camels, the animal and herd prevalences were 1.5% (3/195) (95% CI, 1.4–1.6%) and 2.9% (2/70), (95% CI, 0.9–4.9%), respectively (Table 2), with > 4 mm cut-off. Only one village had reactors (Table 1). In contrast, using the severe method (> 2 mm cut-off), the animal (11.8%; 23/195) and herd (26.8%; 19/70) prevalences were higher and about 56% (9/16) of the villages had at least one reactor camel (Additional files 6 and 7).

### Statistical analysis

In the univariable analysis male camels and male cattle were at approximately 2–3 times at higher risk to be test positive when compared to females (Table 4). Similarly, in the multivariable analysis of the risk factors, only ‘sex’ of the animals remained in the final reduced model, though with borderline significance as shown for the univariable results (Table 4). Overall, male animals had around 3 times higher odds to be test positive than females. The variables ‘species’ and ‘age’, though, apparent potential risk factors, were not statistically significant.

**Table 4:** Species of animals (cattle and camels) and sex as potential risk factors for the presence of BTB reactors at > 4 mm cut-off within the tested animals in the study regions within the extensive traditional livestock husbandry system analyzed by univariable and multivariable logistic regression

Species	*P*-value	OR	95% CI	
			Lower bound	upper bound
Univariable analysis				
Camel versus cattle	0.703	1.3	0.4	4.5
Sex				
Male versus female cattle	0.058	2.9	1.0	8.7
Male versus female camel	0.743	1.5	0.13	16.9
Male versus female (overall)	0.06	2.6	1.0	8.7

### Descriptive epidemiology of the animal and human risk factors on farm level

#### Risk factors for animal BTB exposure

Median numbers of cattle, goats and camels owned per farmer (household) interviewed were 6 (Range:1–208), 11 (Range: 2–250), and 3 (Range: 1–47), respectively. Out of the 232 interviewed households (farmers) keeping cattle, 5.6% (*n* = 13) had positive animals, whereas this was the case in 6.9% (*n* = 2) of camel keeping households. None of the households with goats had SICTT positive animals. Out of the two households with SICTT reactor camels, one also owns dairy farm (MK Ghebremariam, personal experience). Among the interviewed households, 96.6% (*n* = 224) of cattle, 93.8% (*n* = 120) of goat, and 100% (*n* = 29) of camel owners allowed their animals to share watering points with other animals. Besides, 18.1% (*n* = 42) of the cattle, 3.9% (*n* = 5) of the goats, and 13.8% (*n* = 4) of the camel owners reported that they bought and introduced new animals to their existing herds in the last 2–3 years (Table 5). Likewise, 22.0% (*n* = 51) of the cattle, 10.2% (*n* = 13) of the goat, and 55.2% (*n* = 16) of the camel owners reported that their animals migrate to other regions during the dry seasons (Table 5).

**Table 5:** Risk factors for the presence of SICTT reactors as compared between cattle, goats and camels in the extensive livestock husbandry system within the study regions in Eritrea

Variables	Cattle herds (*n* = 232)	Goat herds (*n* = 128)	Camel herds (*n* = 29)	Overall herds (*n* = 389)
	Number (%)	Number (%)	Number (%)	Number (%)
Water point sharing				
Yes	224 (96.6)	120 (93.8)	29 (100)	373 (95.9)
No	8 (3.4)	8 (6.3)	0 (0.0)	16 (4.1)
Introduction of new animals				
Yes	42 (18.1)	5 (3.9)	4 (13.8)	51 (13.1)
No	190 (81.9)	123 (96.1)	25 (86.2)	338 (86.9)
Livestock migration				
Yes	51 (22.0)	13 (10.2)	16 (55.2)	80 (20.6)
No	181 (78.0)	115 (89.8)	13 (44.8)	309 (79.4)
Source of water				
Outside farms	128 (55.2)	46 (35.9)	11 (37.9)	185 (47.6)
Inside farms	49 (21.1)	60 (46.9)	17 (58.6)	126 (32.4)
Inside and outside farms	55 (23.7)	22 (17.2)	1 (3.4)	78 (20.0)

Of the farmers interviewed 38.4% (*n* = 89) reported that their cattle spend the nights in the open including night grazing, 52.7% (*n* = 106) in separate animal houses, 13.8% (*n* = 14) in enclosures, and 2.3% (*n* = 5) share houses with humans at nights (Table 6). They also indicated that 74.2% (*n* = 95) farmers house their goats in separate animal houses, whereas 23.4% (*n* = 30), and 2.3% (*n* = 3), keep them in ‘enclosures made of thorns’ and ‘free roaming in compounds’ at night, respectively (Table 6).

**Table 6:** Housing of cattle and goats at night in Debub (in central highlands with high altitude and mild temperature), Anseba (partially in the central highlands and partially in the lowlands with hot and arid climate), Gash Barka (in the western low lands with hot and arid climate) and Southern Red Sea (in Eastern low land with hot and arid climate) regions within the extensive livestock husbandry system

Variables	Debub (*n* = 106)	Anseba (*n* = 61)	Gash Barka (*n* = 64)	Southern Red Sea (*n* = 1)	Overall (*n* = 232)
Cattle housing	Number (%)	Number (%)	Number (%)	Number (%)	Number (%)
Open area	9 (8.5)	32 (52.5)	48 (75.0)	0 (0.0)	89 (38.4)
Separate animal houses	95 (89.6)	8 (13.1)	3 (4.7)	0 (0.0)	106 (52.7)
Enclosures	0 (0.0)	18 (29.5)	13 (20.3)	1 (100.0)	32 (13.8)
Share houses with humans	2 (1.9)	3 (4.9)	0 (0.0)	0 (0.0)	5 (2.2)
Goat housing	*n* = 39	*n* = 17	*n* = 39	*n* = 33	*n* = 128
Open area	0 (0.0)	0 (0.0)	3 (7.7)	0 (0.0)	3 (2.3)
Separate animal houses	39 (100)	6 (35.3)	19 (48.7)	31 (93.9)	95 (74.2)
Enclosures	0 (0.0)	11(64.7)	17 (43.6)	2 (6.1)	30 (23.4)

#### Risk factors for human BTB exposure

Out of the farmers interviewed, 24.6% (*n* = 57) of cattle, 51.7% (*n* = 15) of camel, and 38% (*n* = 49) of goat owners indicated that raw milk and milk products are consumed in their households regularly (Table 7). Moreover, 4.7% (*n* = 11) of the cattle, and 2.3% (*n* = 33) of the goat owners reported the presence of respiratory diseases in their family members. Of these, one cattle herd was positive with > 4 mm cut-off and two with > 2 mm cut-off. None of goats’ farms that reported the presence of tuberculosis in their families had positive animals. The questionnaires did not include this variable with reference to camels. Among the total number of farmers interviewed, 57.6% (*n* = 224) indicated that BTB awareness campaigns had never been held in their areas. Of the respondents, 42.9% (*n* = 167) had no education (cannot read and write), 37.5% (*n* = 146) had a low (literate; primary level education; grade 1–5), 17.5% (*n* = 68) medium (grade 6–8), and 2.1% (*n* = 8) higher levels of education (grade 9–12/college level education) (Table 7).

**Table 7:** Bovine tuberculosis awareness, education levels, and raw milk consumption habit among farmers keeping cattle, goats and camels, within the traditional livestock farming system in Eritrea

Variables	Cattle owners (*n* = 232)	Goats owners (*n* = 128)	Camel owners (*n* = 29)	Overall (*n* = 389)
TB awareness	Number (%)	Number (%)	Number (%)	Number (%)
Yes	109 (47.0)	46 (35.9)	10 (34.5)	165 (42.4)
No	123 (53)	82 (64.1)	19 (65.5)	224 (57.6)
BTB animal to humans				
Yes	151 (65.1)	91 (71.1)	13 (44.8)	255 (65.6)
No	24 (10.4)	9 (7.0)	10 (34.5)	43 (11.0)
I don’t know	57 (24.7)	28 (21.9)	6 (20.7)	91 (23.4)
TB human to animals				
Yes	23 (9.9)	22 (17.2)	5 (17.2)	50 (12.9)
No	79 (34.1)	37 (28.9)	15 (51.7)	131 (33.7)
I don’t know	130 (56.0)	69 (53.9)	9 (31.0)	208 (53.5)
Level of education				
No education (cannot read and write)	93 (40.1)	53 (41.4)	21 (72.4)	167 (42.9)
Low (literate; grade 1–5 of formal education)	87 (37.5)	56 (43.8)	3 (10.3)	146 (37.5)
Medium (grade 6–8 of formal education)	46 (19.8)	18 (14.1)	4 (13.8)	68 (17.5)
Higher (grade 9–12 formal /college level)	6 (2.6)	1 (0.8)	1 (3.4)	8 (2.1)
Raw milk consumption				
Yes	57 (24.6)	31 (24.2)	25 (86.2)	113 (29.0)
No	175 (75.4)	97 (75.8)	4 (13.8)	276 (71.0)

## Discussion

This study presents the first efforts to assess the prevalence of BTB in cattle, goats and camels, and its zoonotic potential within the extensive traditional livestock husbandry system in Eritrea (Fig. 1). Focusing on cattle, our study reports low (1.2%) individual animal and 3.2% herd prevalences of BTB. Similar findings for individual animal prevalence were reported in Ethiopia, Gumi et al. [21] 5.5%, Ameni et al. [23] 1.8% and 4.7%, Ameni et al. ([24, 25], reported) 7.9% -11.6%, Tschopp et al. ([26, 27], 0.8%; 0.9%), and Mamo et al. [18] reported 11% and a herd prevalence of 44% in Ethiopia in the Afar region with similar conditions as the pastoral areas in our study. In Ghana 13.8% of individual animal prevalence was observed [28]. Although low, the prevalence in our study differed between regions, Debub showing the highest prevalence, 2.3%, where also a high (7.3%) BTB prevalence in dairy cattle was recorded in our previous study [14]. Contact between the dairy cattle and cattle within the extensive system may be postulated as a potential risk factor for the transmission of BTB. This region, located in the central highland of Eritrea, where mixed crop-livestock farming is conducted, is endowed with relative mild temperature and higher precipitation, hence environmental conditions more favourable for survival of *M. bovis* as compared with the arid and semi-arid regions of Gash Barka, Southern Red Sea and Anseba (partially) [14, 29]. In the latter areas, climatic conditions, lower cattle density, housing of animals in open areas (Table 6) may explain the low prevalence of BTB in the extensive cattle production system in general. In our current study, in Anseba region, BTB prevalence was very low (0.5%) in cattle within the extensive livestock production system (Tables 1 and 2). In contrast to Debub region, in Anseba, very low (0.2%) BTB prevalence was reported in dairy cattle [14], thus, in this case, transmission of BTB from the dairy cattle to the indigenous cattle within the extensive farming may be less likely when compared to Debub region.

Although the observed BTB prevalence was low, it is noteworthy that the presence of infection was indicated in many of the study areas (Table 1 and Fig. 1). This may suggest that BTB was introduced to these areas sporadically from various sources but spread was limited. This can be explained by investigating the generally accepted drivers of BTB prevalence, i.e. breed of cattle, farming system (intensive/ extensive), housing and gathering of animals at grazing and watering points. Our current study was conducted exclusively in indigenous (zebu) cattle which are considered relatively resistant to BTB as compared to exotic breeds [13, 25, 30]. Likewise, the extensive livestock management practiced in our study areas is known to pose a far lower risk for BTB progression and transmission than the intensive dairy farming system. It can be argued that under these circumstances and in combination with the prevailing climatic conditions, the risk for BTB transmission is effectively reduced as evidenced by the current low prevalence.

Nevertheless, the existing potential for spread of BTB due to inter-species herd mixing at water points and resting areas where livestock congregate and as well as due to migration and uncontrolled livestock movement must not be underestimated, nor ignored (Table 5). On the other hand, it must be kept in mind that several host related factors like malnutrition, recent infection with *M. bovis*, co-infection with non-tuberculous mycobacteria, infestation with gastrointestinal parasites, and generalized tuberculosis [31–34] are able to decrease reactivity to the SICTT and cannot be ruled out to have influenced our study outcome.

In goats, no positive reactors were found in our current study, and this finding was in agreement with what was reported by Tschopp et al. (2011 and 2010b) [10, 27]. A similar stud in Ethiopia reported 0.5% in small ruminants, [35]. The absence of BTB positive goats in our current study (mixed crop-livestock, and pastoral systems) might be attributed to the restriction of grazing of the flocks within their villages or housing of goats separately from cattle and camels at night. In addition, separate herding may have contributed to a low contact rate between goats and the other species of animals, unlike in the neigbouring Ethiopia where there is congregation and interspecies as well as wildlife mixing in grazing areas [18]. Infestation with liver flukes (*Fasciola hepatica*) and other helminths in the relatively wet highlands and arid low land areas are commonly encountered in slaughtered goats ([36], Tsegay Ghebremeriam, senior meat inspector, MOA, personal communication, and MK Ghebremariam, personal experience) perhaps causing reduced reactivity to the SICTT as reported by several studies [31–33]. In addition, the sensitivity of SICTT in goats, as in cattle, might be compromised by co-infections with viral diseases in chronic stages such as peste des petits ruminants (PPR), Contagious Caprine Pleuropneumonia (CCPP); or sheep and goat pox (MK Ghebremariam, personal experience). In general, the possibilities for goats to become infected are less, as they are browsers and rarely graze pastures that may have been contaminated. Studies also suggest that small ruminants are only spillover hosts that cannot maintain the disease in a herd [37], unless they are in close contact with cattle with high BTB prevalence [9, 38] or managed under intensive production system [39–41]. There is no information on the status of paratuberculosis (*M. avium subspecies paratuberculosis*) in Eritrea, that is known to interfere with the skin test when present [23, 42]. Though our current study was not able to show the presence of BTB in goats, several studies in Africa and Europe with different as well as similar agricultural settings as in Eritrea showed the presence of *M. bovis* and *M. caprae* in goats [1–4, 35, 39–45] thus we need to approach the current finding cautiously since only the OIE standard was used to interpret the results. In indigenous (zebu) cattle as well as in goats, the severe (> 2 mm cut-off) method showed better sensitivity without affecting the specificity of the SICTT as compared to the standard method [21, 44–47]. The use of severe method in our study might have increased the sensitivity of the test. Results that compare the number of positive animals when both the standard (> 4 mm cut-off) and the severe (> 2 mm cut-off) methods are used are presented in additional files (Additional files 6 and 7). Might be good to emphasize the increase spatial spread in case of the severe interpretation/ implying increase risk for infection of animals and humans.

Our study has shown that camels were more at risk of being SICTT reactors as compared with cattle (Table 4), though the association was not significant as only few were positive. BTB is prevalent in dairy cattle in Anseba region, as reported by Ghebremariam et al. [14]. The overall individual animal prevalence in camels was 1.5%. This is considered low as compared with similar studies in Ethiopia and Kenya that showed 6% (29/480) and 37% (15/41) prevalences, respectively, based on standard interpretation [48, 49]. Relatively, the prevalence was higher in the Anseba region (1.5%; Table 2) when compared to other regions of Eritrea where the study was conducted. In this region mixing of camels and cattle is relatively common, and in some cases camel owners also own dairy farms and use their camels to transport animal feed to the farms that may allow camel-cattle contact. Such interactions may have contributed to the presence of more SICTT reactor camels in Anseba as compared with the other study regions. Camels in close contact with cattle were found to be more prone to *M. bovis* infection and to have more tuberculosis lesions in the abattoir than those not having contact with cattle [5, 6, 50–52]. No reactor camel was found in Gash Barka and Southern Red Sea regions. In these regions camels are herded separately from cattle, but trekked long distances where they may come into contact with other animal species en route and at water points. The low prevalence of BTB in camels in the lowlands can be understood as they are browsers, in addition to being herded separately from cattle in a region with low prevalence of BTB in cattle. However, there is no information on the status of helminths, paratuberculosis (*M. avium subspecies paratuberculosis*), other non-tuberculous mycobacteria or viral infection in camels in Eritrea, that may interfere with the skin test when present [31–34, 41, 42].

Comparatively, the overall BTB prevalence at animal level in all the tested species and the number of study areas with reactors was highest in Debub (Fig. 1). Out of the nine positive reactor animals in this region, seven were males. As mixed-crop livestock production system is practiced in this region, male cattle are mostly used either as oxen or for mating purposes, and thus kept longer in the herd than females [29].

Gash Barka is the region where approx. 60% of the livestock population is located and it is the destination for all the animals migrating from different regions of the country, especially, during the dry season. Besides, this region shares borders with Sudan and Ethiopia where uncontrolled movement of animals is possible. The low BTB prevalence in this region might be due to the arid and hot climate which is not suitable for the survival of *M. bovis* as it is readily destroyed by direct sunlight under dry condition [13], in contrast to Debub region.

Focusing on the risk factors for human BTB exposure, overall, within the traditional extensive livestock husbandry system in the selected study areas, 29% of the households consume raw, untreated milk (Table 7). Such milk consumption habit might serve as a vehicle for BTB transmission from infected animals to humans as several studies have shown the presence of *M. bovis* in camels’, goats’ and in cows’ milk [1–4, 53]. Moreover, among the interviewed cattle owners, 2.4% share their houses with their cattle (Table 6). Sharing of the same microenvironment and dwelling between humans and animals has been identified as one of the routes of animal-to-human BTB infection or vice versa, mainly in rural areas in developing countries [12]. The presence of tuberculosis within some of the cattle rearing families and their animals warrants suspicion of the presence of *M. bovis* within the animal-human interface in this production system.

BTB in camels and goats was not considered of veterinary concern in Eritrea, thus, so far, no attempt has been made to conduct BTB testing or routine post mortem examinations in the slaughterhouses for the detection of TB-like lesions. The current study indicated the presence of BTB in cattle and camels, and its spread throughout the study regions within the extensive livestock production system in Eritrea, though at a low prevalence. It warrants future, more in-depth, studies on BTB in these livestock species.

Our study has one major limitation, i.e., the number of the farmers that completed the questionnaires were fewer than the animal herds tested. This was mainly attributed to the overcrowding of the testing sites and the hot and arid climate that forced the farmers to leave the testing sites without filling the questionnaires (even after presenting their animals for testing). As the observed level of animal prevalence is low, a multilevel analysis of the data was not estimable although such a model would give more precise estimates if feasible. Such a model also needs sufficient observations within each cluster which was not the case in our study as many very small herds are present, most of the herds consist of 1–3 animals.

## Conclusion and recommendation

The current study has shown that SICTT reactors are rare in cattle and camels and were not found in goats. However, though rare, the spatial distribution of the affected animals across most of the selected regions (Fig. 1), where consumption of unpasteurized milk is common, warrants continuous surveys, cautious and timely control measures of the disease. We recommend the testing of the animals to be conducted during mild weather seasons so as to be able to conduct face-to-face interviews and complete the questionnaires that would match the number of herds tested.

## Additional files

Additional file 1: Regions, sub-regions and villages included in the study, and species, breed, sex and age of each animal tested using the SICTT during the study periods (XLSX 77 kb)

Additional file 2: Questionnaire for BTB risk factors study within the cattle raising communities in the extensive livestock husbandry system in Eritrea. (PDF 333 kb)

Additional file 3: Questionnaire for BTB risk factors study within the goat raising communities in the extensive livestock husbandry system in Eritrea. (PDF 256 kb)

Additional file 4: Questionnaire for BTB risk factors study within the camel raising communities in the extensive livestock husbandry system in Eritrea. (PDF 351 kb)

Additional file 5: Consolidated criteria for reporting qualitative studies (COREQ): 32-item checklist and answers. (DOCX 17 kb)

Additional file 6: Number (and herd) of skin tested cattle, goats and camels, and number (herd) of reactors at region, sub-region and study areas levels using the standard (> 4 mm cut-off) method in the selected study areas within the traditional livestock husbandry system in Eritrea presented for comparison. ‘0’ = zero animals tested from zero herds. NA = not applicable. (DOCX 25 kb)

Additional file 7: BTB prevalence in cattle, goats and camels at individual animal and herd levels within the traditional livestock husbandry system in Eritrea using the standard and severe cut-offs (> 4 mm and > 2 mm) presented for comparison. NA = not applicable. (DOCX 17 kb)

## Abbreviations

BTB: Bovine tuberculosis
ERF: Eritrean Research Fund
FAO: Food and Agriculture Organization
HAC: Hamelmalo Agricultural College
*M. bovis*: *Mycobacterium bovis*
MOA: Ministry of Agriculture
NAPHL: National Animal and Plant Health Laboratory
NUFFIC: Netherlands University Foundation for International Cooperation
OIE: World Organization for Animal Health
PPD: Purified protein derivative
PPD-A: Purified protein derivative for *mycobacterium avium*
PPD-B: Purified protein derivative for *mycobacterium bovis*
SICTT: Single intradermal comparative tuberculin test
