# Supplementary material for: Prevalence of bovine tuberculosis in cattle, goats, and camels of traditional livestock raising communities in Eritrea
Source: BMC Vet Res. 2018 Mar 7;14:73. doi: 10.1186/s12917-018-1397-0 (PMC5842630; doi:10.1186/s12917-018-1397-0)
Supplement: Supplementary file 2 — Questionnaire for BTB risk factors study within the cattle raising communities in the extensive livestock husbandry system in Eritrea. (PDF 333 kb) [file 12917_2018_1397_MOESM2_ESM.pdf]

CATTLE TB RISK ASSESSMENT QUESTIONNAIRE (updated)

SAMPLING INFORMATION (to remain confidential) R/N0._______

1. What is your full name?

2. What is your address?

Zoba:
Sub-zoba:
Kebabi:
Village:

SECTION A follows on the next page ...

Respondents number (R/N0.) ________________________________

Please answer all the questions by circling a number in a shaded box or by

Writing your answer in the shaded space provided.

SECTION A PERSONAL DETAILS

3. What region is being sampled?

Debub	1
Anseba	2
Gash Barka	3
Southern Red Sea	4

V1[ ]

4. What is your age?

V2[ ]

5. What is your highest level of education?

I cannot read and write (Illiterate)	1
I can read and write (nonformal education)	2
Primary School education (grade 1-5)	3
Secondary School education (grade 6-8)	4
High School education (grade 9-12)	5
Tertiary education at a College/University	6

V3[ ]

6. What is your present occupation?

V4[ ]

Unemployed (Pensioner etc.)	1
Self employed	2
Industrial employee	3
Farmer	4
Professional occupation	5
Other (specify :)	

Question 7 follows on the next page ...

7. What is your monthly income which you use to pay your household expenses?

V5[ ]

8. In your household how many of each of the following are present?

Children aged less than 18 yrs.	
Persons older than 18 yrs. and less or equal to 30 yrs.	
Persons older than 30 yrs. and less or equal to 60 yrs.	
Persons older than 60 yrs.	

V6[ ]

SECTION B FARMING DETAILS

9. Which of the following cattle breeds constitute your herd?

Barka	1
Arado	2
Arabo	3
Sudanese cattle (Kenana)	4
Unknown	5
Others (Specify)	

V7[ ]

10. What numbers do you have for the following?

Cows	
Heifers	
Calves	
Bulls	
Oxen	

V8[ ]

11. Did you bring in cattle to your farm during the past 12 months?

Yes	1
No	2

V9[ ]

12. If your answer to Question 11 is “Yes”, where did the cattle come from?

V10[ ]

Question 13 follows on the next page

13. Do your cattle come into contact with any of the following?

Antelopes	1
Greater kudu	2
Warthogs	3
Goats	4
Camels	5
Any other (specify :)	

V11[ ]

14. Where do you water your cattle?

In the river	1
Common watering point (also shared by others)	2
Well water is available at the farm	3
Tap water is available	4
Other (specify):	

V12[ ]

15. Do your cattle share the watering point with other animals?

Yes	1
No	2

V13[ ]

16. If your answer for question 15 is yes. Which other animals share the watering point?

Cattle	1
Goats	2
sheep	3
Camels	4
All	5
Others(specify):	

V14[ ]

17. Have you been aware of signs of respiratory diseases in your herd in the past 6 months?

Yes	1
No	2

V15[ ]

18.If your answer to question number 17 is yes, which of the following clinical signs did you observe?

Respiratory disease (laboured breathing)	1
Coughing	2
Anorexia (lack of appetite)	3
Listlessness	4
Emaciation	5

V16[ ]

19. Have your cattle been tested for tuberculosis in the past 12 months?

Yes	1
No	2

V17[ ]

Question 20 follows on the next page…

20. Who does the herding? V18[ ]

Father	1
Mother	2
Children	3
Hired herder	4
Other (specify)	5

21. Where do you house your animals at night? V19[ ]

Free roaming in the compound	1
In a separate house built for animals	2
In an enclosure made of thorns/stones	3
Share the same house with humans	4
In a hudmo (highland), but separate shade for animals	5
Other (specify)	

22. Do your animals migrate? V20[ ]

Yes	1
No	2

23. If your answer to question number 20 is yes, where to do they migrate to? V21[ ]

24. Name the migration routes (to and from the destination). V22[ ]

25. Indicate the time (season) of migration (months). V23[ ]

SECTION C ZOONOSIS RISK ASSESSMENT

26. What is the average milk yield per cow per day in liters? V24[ ]

Question 27 follows on the next page…

27. What state do you sell your milk to village community members/others?

V25[ ]

I do not sell my milk to community members	1
Fresh (untreated)	2
Boiled	3
Soured	4

28. In your household, which state is your milk consumed and how many in your household consume it?

V26[ ]

Age group consuming your cows' milk		How milk is consumed		
	Fresh (untreated) Boiled		Soured	
			Boiled	untreated
Under 18 yrs.				
18 years to 30 yrs.				
Older than 30 years to 50 yrs.				
Older than 50 years to 70 yrs.				
Older that 70 yrs.				

29. Where do you buy your milk from? Please indicate the type of milk you buy.

V27[ ]

Places where the milk is bought from	Type of milk bought		
	Fresh (untreated)	Boiled	Soured
Shops			
Kiosks			
Tea rooms			
Market places			
Bus stations			
Stalls along the road			
Other (Specify)			

30. Which of the following animal is the source of milk for you (your family)?

V28[ ]

Cattle	1
Goats	2
Camels	3
Others (specify)	

31. Have you ever noticed respiratory disease (TB) in your family?
Or any one of your family diagnosed with respiratory disease (TB)?

V29[ ]

Yes	1
No	2

Section D follows on the next page

Section D TB Awareness campaign

32. What is the status of awareness campaigns about cattle TB in your area?

V30[ ]

There is no awareness campaign in my area	1
Campaign the State Vet. Extension Service	2
Campaign from the State Health Extension Service	3
Other (Specify:)	

V31[ ]

33. Can people contract TB from cattle?

Yes	1
No	2
I don't know	3

V32[ ]

34. Can cattle contract TB from humans?

Yes	1
No	2
I don't know	3

35. Considering your answer to Question 32&33, where did you get your information from?

V33[ ]

I don't know (I can't remember)	1
There is no information available	2
State Vet. Extension Service	3
Human Health Clinic	4
The media	5
Other members of the community	6
Other (specify):	

Thank you for your time and co-operation
