# Supplementary material for: Prevalence of bovine tuberculosis in cattle, goats, and camels of traditional livestock raising communities in Eritrea
Source: BMC Vet Res. 2018 Mar 7;14:73. doi: 10.1186/s12917-018-1397-0 (PMC5842630; doi:10.1186/s12917-018-1397-0)
Supplement: Supplementary file 4 — Questionnaire for BTB risk factors study within the camel raising communities in the extensive livestock husbandry system in Eritrea. (PDF 351 kb) [file 12917_2018_1397_MOESM4_ESM.pdf]

CAMELS TB RISK ASSESSMENT QUESTIONNAIRE (Eritrea)

SAMPLING INFORMATION (to remain confidential) R/N0._______

1. What is your full name?

2. What is your address?

Zoba:
Sub-zoba:
Kebabi:
Village:

SECTION A follows on the next page ...

For official use only

Respondents number (R/N0.) ______________________________

Please answer all the questions by circling a number in a shaded box or by

Writing your answer in the shaded space provided.

SECTION A PERSONAL DETAILS

3. What region is being sampled?

V1[ ]

Debub	1
Anseba	2
Gash Barka	3
Southern Red Sea	4

4. What is your age?

V2[ ]

5. What is your highest level of education?

V3[ ]

I cannot read and write (Illiterate)	1
I can read and write (nonformal education)	2
Primary School education	3
Secondary School education	4
High School education	5
Tertiary education at a College/University	6

6. What is your present occupation?

V4[ ]

Unemployed (Pensioner etc.)	1
Self employed	2
Industrial employee	3
Farmer	4
Professional occupation	5
Other (specify :)	

Question 7 follows on the next page ...

7. What is your monthly income which you use to pay your household expenses?

V5[ ]

8. In your household how many of each of the following are present?

Children aged less than 18 yrs.	
Persons older than 18 yrs. and less or equal to 30 yrs.	
Persons older than 30 yrs. and less or equal to 60 yrs.	
Persons older than 60 yrs.	

V6[ ]

SECTION B FARMING DETAILS

9. Do you own camels?

Yes	1
No	2

V7[ ]

10. What numbers do you have for the following?

<1 year of age	
1-3 years	
>3-5 years	
>5-8 years	
>8 years	

V8[ ]

11. Did you bring in new camels to your herd during the past 12 months?

Yes	1
No	2

V9[ ]

12. If your answer to Question 11 is "Yes", where did the camels come from?

V10[ ]

13. Do your camels come into contact with any of the following?

Antelopes	1
Greater kudu	2
Warthogs	3
Cattle	4
Other camels	5
Goats	6
Sheep	7
Others (specify):	

V11[ ]

14. Where do you water your camels?

In the river	1
Common watering point (also shared by others)	2
Well water is available at the farm	3
Tap water is available	4
Other (specify):	

V12[ ]

15. Do your camels share the watering point with other animals?

Yes	1
No	2

V13[ ]

16. If your answer for question 15 is yes, which other animals share the watering point?

Cattle	1
Other camels	2
sheep	3
goats	4
All	5
Others(specify):	

V14[ ]

17. Do your camels share grazing/browsing areas with other animals?

Yes	1
No	2

V15[ ]

18. If your answer to question 17 is yes, which other animals share the same grazing/browsing areas with your camels?

Cattle	1
Other camels	2
sheep	3
goats	4
All	5
Others(specify):	

V16[ ]

19. Have you been aware of signs of respiratory diseases in your herd in the past 6 months?

Yes	1
No	2

V17[ ]

20.If your answer to question number 17 is yes, which of the following clinical signs did you observe?

Respiratory disease (laboured breathing)	1
Coughing	2
Anorexia (lack of appetite)	3
Listlessness	4
Emaciation	5

V18[ ]

21. Have your camels ever been tested for tuberculosis?

Yes	1
No	2

V19[ ]

V20[ ]

22. If your answer to question 19 is "yes", when was that?

6 months ago	1
1 year ago	2
2 – 3 years ago	3
I cannot remember	4

V21[ ]

23. Do your camels migrate?

Yes	1
No	2

24. If your answer to question number 20 is "yes", where to do they migrate**?**

V22[ ]

25. When do they migrate?

23[ ]

Months	From	To

26. Name the migration routes (to and from the destination).

24[ ]

From your village:
To your village:

SECTION C ZOONOSIS RISK ASSESSMENT

25[ ]

27. Why do you keep camels?

For milk	1
For meat	2
For cash	3
Milk and meat	4
All	5
Other (specify)	6

28. What is the average milk yield per cow (camel) per day in litres? V26[ ]

29. What state do you sell your milk to village community members/others? V27[ ]

I do not sell my milk to community members	1
Fresh (untreated)	2
Boiled	3
Soured	4

30. In your household, which state is your milk consumed and how many in your household consume it? V28[ ]

Age group consuming your cows' milk		How milk is consumed		
	Fresh	Boiled	Soured	
Under 18 yrs.			Fresh	Boiled
18 years to 30 yrs.				
Older than 30 years to 50 yrs.				
Older than 50 years to 70 yrs.				
Older that 70 yrs.				

31. Where do you buy your milk from? Please indicate the type of milk bought? V29[ ]

Places where the milk is bought from	Type of milk bought			
	Fresh	Boiled	Soured	
			Fresh	Boiled
Shops	1	2	3	4
Tea rooms	1	2	3	4
Market places	1	2	3	4
Bus stations	1	2	3	4
Stalls along the road	1	2	3	4
Other (Specify:)				

32. Which of the following animal is the source of milk for you (your family)? V30[ ]

Cattle	1
Camels	2
Camels	3
Others (specify)	

33. What is the status of awareness campaigns about camel TB in your area? V31[ ]

There is no awareness campaign in my area	1
Campaign the State Vet. Extension Service	2
Campaign from the State Health Extension Service	3
Other (Specify:)	

34. Can people contract TB from camels?

Yes	1
No	2
I don't know	3

V32[ ]

35. Can camels contract TB from infected people?

Yes	1
No	2
I don't know	3

V33[ ]

36. Considering your answer to Question 30 & 31 , where did you get your information from?

I don't know (I can't remember)	1
There is no information available	2
State Vet. Extension Service	3
Human Health Clinic	4
The media	5
Other members of the community	6
Other (specify):	

V34[ ]

Thank you for your time and co-operation
