# Supplementary material for: Prevalence of bovine tuberculosis in cattle, goats, and camels of traditional livestock raising communities in Eritrea
Source: BMC Vet Res. 2018 Mar 7;14:73. doi: 10.1186/s12917-018-1397-0 (PMC5842630; doi:10.1186/s12917-018-1397-0)
Supplement: Supplementary file 5 — Consolidated criteria for reporting qualitative studies (COREQ): 32-item checklist and answers. (DOCX 17 kb) [file 12917_2018_1397_MOESM5_ESM.docx]

| **Checklist** | **Answers** |
| --- | --- |
| **Domain 1: Research team and reflexivity** |  |
| **Personal Characteristics** |  |
| 1. Interviewer/facilitator: Which author/s conducted the interview or focus group? | 1^st^ author |
| 2. Credentials: What were the researcher’s credentials? E.g. PhD, MD | DVM, MSc, PhD fellow (1st author), DVM/PhDs, MSc |
| 3. Occupation: What was their occupation at the time of the study? | Asst. professor and researcher in Department of Veterinary Sciences (1^st^ author), professors, statistician |
| 4. Gender: Was the researcher male or female? | Males and females |
| 5. Experience and training What experience or training did the researcher have? | Training in epidemiology (in both veterinary and human medicine), research methodology, pathology, diagnosis, etc |
| **Relationship with participants** |  |
| 6. Relationship established: Was a relationship established prior to study commencement? | Farmers were informed about the study and its objectives through Ministry of Agriculture offices, regional and sub-regional leaders and village elders |
| 7. Participant knowledge of the interview |  |
| What did the participants know about the researcher? e.g. personal goals, reasons for doing the research | Participants were aware about the study and the objectives of the research before the start of the study |
| 8. Interviewer characteristics: What characteristics were reported about the interviewer/facilitator? e.g. Bias, assumptions, reasons and interests in the research topic | NA |
| Domain 2: study design |  |
| **Theoretical framework** |  |
| 9. Methodological orientation and Theory |  |
| What methodological orientation was stated to underpin the study? e.g. grounded theory, discourse analysis, ethnography, phenomenology, content analysis | Content analysis |
| **Participant selection** |  |
| 10. Sampling: How were participants selected? e.g. purposive, convenience, consecutive, snowball | Convenience sampling for testing the animals and purposive sampling for the face-to-face interview. |
| 11. Method of approach How were participants approached? e.g. face-to-face, telephone, mail, email | Face-to-face interview |
| 12. Sample size: How many participants were in the study? | 389 heads of households |
| 13. Non-participation: How many people refused to participate or dropped out? Reasons? | 339 heads of households did not participate in the interview. The reason for non-participation was due to the hot weather that couldn’t allow the farmers to stay longer, and due to their being animal herders that urged the farmers to move from place to place and partially due to our purposive sampling. |

**Additional file 5.**

| **Setting** |  |
| --- | --- |
| 14. Setting of data collection: Where was the data collected? e.g. home, clinic, workplace | Interviews were conducted at water points, fields and homes |
| 15. Presence of non-participants: Was anyone else present besides the participants and researchers? | Yes, staff members of the veterinary services who were assisting during the testing were present. |
| 16. Description of sample: What are the important characteristics of the sample? e.g. demographic data, date | Demographic, sharing of households with animals, consumption of unpasteurized milk, educational level, level of BTB awareness |
| **Data collection** |  |
| 17. Interview guide: Were questions, prompts, guides provided by the authors? Was it pilot tested? | Closed(mainly) and open questions were provided (Semi-structured questionnaire). The questionnaire was pilot tested. |
| 18. Repeat interviews: Were repeat interviews carried out? If yes, how many? | No repeat interview was conducted. |
| 19. Audio/visual recording: Did the research use audio or visual recording to collect the data? | No |
| 20. Field notes: Were field notes made during and/or after the interview or focus group? | The filed notes were made during the interview. |
| 21. Duration: What was the duration of the interviews or focus group? | The duration of the interview was 20-25 minutes |
| 22. Data saturation: Was data saturation discussed? | There was no data saturation. The interview was face-to-face |
| 23. Transcripts returned: Were transcripts returned to participants for comment and/or correction? | No transcripts were returned to participants. |
| **Domain 3: analysis and findings** |  |
| **Data analysis** |  |
| 24. Number of data coders: How many data coders coded the data? | One person only. Coding were conducted for easy of data analysis, otherwise the participants own meaning and perspectives are presented in the report. |
| 25. Description of the coding tree: Did authors provide a description of the coding tree? | No |
| 26. Derivation of themes: Were themes identified in advance or derived from the data? | Yes |
| 27. Software: What software, if applicable, was used to manage the data? | SPSS, and Microsoft Excel |
| 28. Participant checking: Did participants provide feedback on the findings? | NA |
| **Reporting** |  |
| 29. Quotations presented: Were participant quotations presented to illustrate the themes / findings? Was each quotation identified? e.g. participant number | NA |
| 30. Data and findings consistent: Was there consistency between the data presented and the findings? | Yes |
| 31. Clarity of major themes: Were major themes clearly presented in the findings? | Yes |
| 32. Clarity of minor themes: Is there a description of diverse cases or discussion of minor themes? | Yes |

Additional file 5 (cont...)
