# Supplementary material for: Prevalence of bovine tuberculosis in cattle, goats, and camels of traditional livestock raising communities in Eritrea
Source: BMC Vet Res. 2018 Mar 7;14:73. doi: 10.1186/s12917-018-1397-0 (PMC5842630; doi:10.1186/s12917-018-1397-0)
Supplement: Supplementary file 6 — Number (and herd) of skin tested cattle, goats and camels, and number (herd) of reactors at region, sub-region and study areas levels using the standard (> 4 mm cut-off) method in the selected study areas within the traditional livestock husbandry system in Eritrea presented for comparison. ‘0’ = zero animals tested from zero herds. NA = not applicable. (DOCX 25 kb) [file 12917_2018_1397_MOESM6_ESM.docx]

**Additional file 6.**

| **Regions** | **Sub-region** | **Study Areas** | **Tested cattle(herd)** | **Reactor cattle (herd)** | | **Tested goats (herds)** | **Reactor goats (herd)** | | **Tested camels (herds)** | **Reactor camels (herd)** | |
| --- | --- | --- | --- | --- | --- | --- | --- | --- | --- | --- | --- |
|  |  |  |  | **>4mm** | **>2mm** |  | **>4mm** | **>2mm** |  | **>4mm** | **>2mm** |
| **Debub** | **Dbarwa** | DRW1 | 61(20) | 0(0) | 3(3) | 60(13) | 0(0) | 0(0) | NA | NA | NA |
|  |  | DRW2 | 44(29) | 3(3) | 8(7) | 15(6) | 0(0) | 0(0) | NA | NA | NA |
|  |  | DRW3 | 38(26) | 0(0) | 0(0) | 17(7) | 0(0) | 0(0) | NA | NA | NA |
|  |  | DRW4 | 48(15) | 0(0) | 0(0) | 25(6) | 0(0) | 0(0) | NA | NA | NA |
|  | **Total** |  | **191 (90)** | **3(3)** | **11(10)** | **117 (32)** | **0(0)** | **0(0)** | **NA** | **NA** | **NA** |
|  | **Mendefera** | MFR1 | 104(35) | 4(4) | 14 (12) | 38(7) | 0(0) | 0(0) | NA | NA | NA |
|  |  | MFR22 | 52(23) | 1(1) | 3(3) | 16(5) | 0(0) | 0(0) | NA | NA | NA |
|  |  | MFR3 | 38(15) | 1(1) | 2(2) | 0(0) | - | - | NA | NA | NA |
|  | **Total** |  | **194 (73)** | **6(6)** | **19(17)** | **54(12)** | **0(0)** | **0(0)** | **NA** | **NA** | **NA** |
| **Anseba** | **Hagaz** | HAZ1 | 3(2) | 0(0) | 1(1) | 15(2) | 0(0) | 3(1) | 0 | - | - |
|  |  | HAZ2 | 12(2) | 0(0) | 1(1) | 10(1) | 0(0) | 2 (1) | 1(1) | 0(0) | 1(1) |
|  |  | HAZ3 | 3(1) | 0(0) | 0(0) | 0 | - | - | 0 | - | - |
|  |  | HAZ4 | 8(2) | 0(0) | 0(0) | 0 | - | - | 0 | - | - |
|  |  | HAZ5 | 0 | - | - | 13(2) | 0(0) | 5(2) | 10(1) | 0(0) | 0(0) |
|  |  | HAZ6 | 0 | - | - | 3(1) | 0(0) | 0(0) | 0 | - | - |
|  |  | HAZ7 | 0 | - | - | 5(1) | 0(0) | 0(0) | 0 | - | - |
|  | **Total** |  | **26(7)** | **0(0)** | **2(2)** | **46(7)** | **0(0)** | **10(4)** | **11(2)** | **0(0)** | **1(1)** |
|  | **Hamelmalo** | HAM1 | 14(6) | 0(0) | 0(0) | 0 | 0(0) | 0(0) | 0 | - | - |
|  |  | HAM2 | 1(1) | 0(0) | 0(0) | 0 | 0(0) | 0(0) | 0 | - | - |
|  |  | HAM3 | 61(27) | 0(0) | 1(1) | 37(7) | 0(0) | 0(0) | 11(4) | 3(2) | 4(2) |
|  |  | HAM4 | 45(11) | 0(0) | 1(1) | 11(2) | 0(0) | 0(0) | 0 | - | - |
|  |  | HAM5 | 0(0) | - | - | 0(0) | - | - | 0 | - | - |
|  | **Total** |  | **121(45)** | **0(0)** | **2(2)** | **48(9)** | **0(0)** | **0(0)** | **10(3)** | **3(2)** | **4(2)** |
|  | **Adi-Tekelezan** | ATK1 | 72(35) | 1(1) | 3(3) | 39(9) | 0(0) | 0(0) | 0 | - | - |
|  | **Total** |  | **72(35)** | **1(1)** | **3(3)** | **39(9)** | **0(0)** | **0(0)** | **0** | **-** | **-** |

| **Regions** | **Sub-region** | **Study Areas** | **Tested cattle(herd)** | **Reactor cattle (herd)** | | **Tested goats (herds)** | **Reactor goats (herd)** | | **Tested camels (herds)** | **Reactor camels (herd)** | | |
| --- | --- | --- | --- | --- | --- | --- | --- | --- | --- | --- | --- | --- |
|  |  |  |  | **>4mm** | **>2mm** |  | **>4mm** | **>2mm** |  | | **>4mm** | **>2mm** |
| **Gash Barka** | **Barentu** | BAR1 | 89(32) | 1(1) | 5(4) | 0 | - | - | 0 | | - | - |
|  |  | BAR2 | 28(6) | 0 (0) | 0 (0) | 7(2) | 0(0) | 0(0) | 0 | | - | - |
|  |  | BAR3 | 6(4) | 0(0) | 0(0 | 49(13) | 0(0 | 0(0 | 13(10) | | 0(0) | 3(3) |
|  |  | BAR4 | 22(10) | 0 (0) | 1(1) | 72(18) | 0(0) | 4(3) | 2(2) | | 0 (0) | 1(1) |
|  | **Total** |  | **145(52)** | **1(1)** | **6(5)** | **128(33)** | **0(0)** | **4(3)** | **15(12)** | | **0(0)** | **4(4)** |
|  | **Tessenei** | TES1 | 28(12) | 0 (0) | 2(2) | 163(70) | 0(0 | 2(2) | 1(1) | | 0 (0) | 1(1) |
|  |  | TES2 | 11(4) | 0 (0) | 1(1) | 22(9) | 0(0) | 0(0) | 0 | | - | - |
|  |  | TES3 | 0 | - | - | 0 | - | - | 71(20) | | 0 (0) | 5 (4) |
|  |  | TES4 | 30(10) | 0 (0) | 1(1) | 55(21) | 0(0) | 2(2) | 1(1) | | 0 (0) | 0 (0) |
|  |  | TES5 | 15(5) | 0(0) | 0(0) | 10(5) | 0(0) | 0(0) | 0(0) | | - | - |
|  | **Total** |  | **84(31)** | **0(0)** | **4(4)** | **250(105)** | **0(0)** | **4(4)** | **73(22)** | | **0(0)** | **6(5)** |
|  | **Hykota** | HYK1 | 17(5) | 0(0) | (0) | 0 | - | - | 0 | | - | - |
|  |  | HYK2 | 16(8) | 0 (0) | 2(2) | 0 | - | - | 1(1) | | 0 (0) | 0 (0) |
|  |  | HYK3 | 11(11) | 1(1) | 2(2) | 0 | - | - | 10(2) | | 0(0) | 0(0) |
|  |  | HYK4 | 8(1) | 0(0) | 0(0) | 0 | - | - | 0 | | - | - |
|  |  | HYK5 | 28(8) | 0 (0) | 1(1) | 0 | - | - | 0 | | - | -) |
|  |  | HYK6 | 14(14) | 0 (0) | 0 (0) | 0 | - | - | 48 (6) | | 0 (0) | 2(2) |
|  |  | HYK7 | 31(1) | 0(0) | 1(1) | 0 | - | - | 0 | | - | - |
|  |  | HYK8 | 28(1) | 0(0) | 0(0) | 0 | - | - | 0 | | - | - |
|  | **Total** |  | **153(49)** | **1(1)** | **6(6)** | **0** | **-** | **-** | **59(9)** | | **0(0)** | **2(2)** |
|  | **Mogolo** | MOG1 | 46(9) | 1(1) | 1(1) | 25(2) | 0(0) | 0(0) | 11(8) | | 0 (0) | 5(4) |
|  | **Total** |  | **46(9)** | **1(1)** | **1(1)** | **25(2)** | **0(0)** | **0(0)** | **11(8)** | | **0(0)** | **5(4)** |
|  | **Akurdet** | AKU1 | 25(15) | 0 (0) | 5(3) | 0 | - | - | 0 | | - | - |
|  |  | AKU2 | 18(5) | 0(0) | 0(0) | 0 | - | - | 0 | | - | - |
|  | **Total** |  | **43(20)** | **0(0)** | **5(3)** | **0(** | **-** | **-** | **0** | | **-** | **-** |
| **Southern Red Sea** | **Debub Dankalia** | DANK1 | 0 | - | - | 17(4) | 0(0) | 0(0) | 3(3) | | 0 (0) | 0(0) |
|  |  | DANK2 | 0 | - | - | 10(1) | 0(0) | 0(0) | 2(2) | | 0 (0) | 0 (0) |
|  |  | DANK3 | 0 | - | - | 54(13) | 0(0) | 1 (1) | 0 | | - | - |
|  |  | DANK4 | 2(2) | 0(0) | 0(0) | 35(10)) | 0(0) | 0(0) | 0 | | - | - |
|  |  | DANK5 | 0 | - | - | 53(6) | 0(0) | 0(0) | 11(9) | | 0(0) | 1(1) |
|  | **Total** |  | **2(2)** | **0(0)** | **0(0)** | **169(34)** | **0(0)** | **1(1)** | **16(14)** | | **0(0)** | **1(1)** |
|  | **Grand Total** |  | **1077(413)** | **13(13)** | **59(53)** | **876(243)** | **0(0)** | **19(12)** | **195(70)** | | **3(2)** | **23(19)** |

**Additional file 6.** (cont…)
