# Supplementary material for: Prevalence of bovine tuberculosis in cattle, goats, and camels of traditional livestock raising communities in Eritrea
Source: BMC Vet Res. 2018 Mar 7;14:73. doi: 10.1186/s12917-018-1397-0 (PMC5842630; doi:10.1186/s12917-018-1397-0)
Supplement: Supplementary file 7 — BTB prevalence in cattle, goats and camels at individual animal and herd levels within the traditional livestock husbandry system in Eritrea using the standard and severe cut-offs (> 4 mm and > 2 mm) presented for comparison. NA = not applicable. (DOCX 17 kb) [file 12917_2018_1397_MOESM7_ESM.docx]

Additional file 7.

| **Number and herds of cattle, goats and camels tested** | | **Anseba** | **Debub** | **Gash Barka** | **Southern Red Sea** | **Overall** |
| --- | --- | --- | --- | --- | --- | --- |
| Number (%) of cattle | | 219 (20.3) | 385 (35.7) | 471 (43.7) | 2 (0.2) | 1077 (100) |
| Number (%) of goats | | 133 (15.2) | 171 (19.5) | 403 (46) | 169 (19.3) | 876 (100) |
| Number (%) of camels | | 21 (10.8) | NA | 158 (81.0) | 16 (8.2) | 195 (100) |
| **Total number (%) tested/region** | | **373 (17.4)** | **556 (25.9)** | **1032 (48)** | **187 (8.7)** | **2148 (100)** |
| **Individual animal Prevalence (%)** | |  |  |  |  |  |
| **Cattle** | 4mm cut-off | 1 (0.5) | 9 (2.3) | 3 (0.6) | 0 (0.0) | 13 (1.2) |
|  | 2mm cut-off | 7 (3.2) | 30 (7.8) | 22 (4.7) | 0 (0.0) | 59 (5.5) |
| **Goats** | 4mm cut-off | 0 (0.0) | 0 (0.0) | 0 (0.0) | 0 (0.0) | 0 (0.0) |
|  | 2mm cut-off | 10 (7.5) | 0 (0.0) | 8 (2.0) | 1 (0.6) | 19 (2.2) |
| **Camels** | 4mm cut-off | 3 (13.6) | NA | 0 (0.0) | 0 (0.0) | 3 (1.5) |
|  | 2mm cut-off | 5 (21.7) | NA | 17 (10.8) | 1 (6.3) | 23 (11.8) |
| **Herds of cattle, goats and camels tested** | |  |  |  |  |  |
| Herds (%) of cattle | | 87 (21.1) | 163 (39.5) | 161 (38.9) | 2 (0.5) | 413 (100) |
| Herds (%) of goats | | 25 (10.3) | 44 (18.1) | 140 (57.6) | 34 (14.0) | 243 (100) |
| Herds (%) of camels | | 6 (8.3) | 0 (0.0) | 50 (72.2) | 14 (19.4) | 70 (100) |
| **Herd prevalence (%)** |  |  |  |  |  |  |
| **Cattle** | 4mm cut-off | 1 (1.2) | 9 (5.5) | 3 (1.9) | 0 (0.0) | 13 (3.2) |
|  | 2mm cut-off | 7 (8.1) | 28 (17.2) | 19 (11.8) | 0 (0.0) | 54 (13.1) |
| **Goats** | 4mm cut-off | 0 (0.0) | 0 (0.0) | 0 (0.0) | 0 (0.0) | 0 (0.0) |
|  | 2mm cut-off | 4 (16) | 0 (0.0) | 7 (5.1) | 1 (2.9) | 12 (5.0) |
| **Camels** | 4mm cut-off | 2 (33.3) | NA | 0 (0.0) | 0 (0.0) | 2 (2.9) |
|  | 2mm cut-off | 3 (50.0) | NA | 15 (30) | 1 (7.1) | 19 (27.1) |
